# Blue energy recovery in the Atacama Desert using electrochemical ion pumping devices: a Chilean perspective on salinity gradient energy

**DOI:** 10.3389/fchem.2025.1659479

**Published:** 2025-08-19

**Authors:** Felipe M. Galleguillos Madrid, Sebastián Salazar-Avalos, Markus Bergendahl, Javier Quispe, Norman Toro, Galvarino Casanueva-Yáñez, Alvaro Soliz

**Affiliations:** ^1^ Centro de Desarrollo Energético Antofagasta, Universidad de Antofagasta, Antofagasta, Chile; ^2^ Departamento de Ingeniería Química y Medio Ambiente, Universidad Católica del Norte, Antofagasta, Chile; ^3^ Faculty of Engineering and Architecture, Universidad Arturo Prat, Iquique, Chile; ^4^ Facultad de Ingeniería y Negocios Universidad de Las Américas, Santiago, Chile; ^5^ Departamento de Ingeniería en Metalurgia, Universidad de Atacama, Copiapó, Chile

**Keywords:** blue energy, Mixing Entropy Battery (MEB), salinity gradient energy, electrochemical ion pumping, Atacama Desert, Atacama brine

## Abstract

The growing global demand for clean and sustainable energy has intensified the development of novel technologies capable of harnessing naturally available resources. Among these, blue energy, referring to the power generated from the mixing of waters with different salinities, has emerged as a promising yet underutilized source. This perspective presents a comprehensive synthesis of recent advances in electrochemical harvesting systems, with a particular focus on Mixing Entropy Batteries (MEBs) as efficient, membrane-free devices for salinity gradient energy recovery. Unlike conventional approaches such as Reverse Electrodialysis (RED) and Pressure Retarded Osmosis (PRO), which depend heavily on ion-exchange membranes and complex infrastructure, MEBs offer simplified and scalable architecture suitable for harsh environments and industrial effluents. The use of LiCl-based electrolytes enables significant blue energy recovery, achieving energy densities of 38.2 mJ/cm^2^ and power densities of 13.8 μW/cm^2^, with excellent cycling stability. This system leverages the high solubility of LiCl (832 g/L) to create steep salinity gradients, utilizing LiFePO_4_/FePO_4_ as the cathode and Ag/AgCl as the anode, with no observable performance degradation over 100 cycles. This work analyzes alternative electrode materials, including Prussian Blue analogues (copper hexacyanoferrate CuHCF), MnO_2_, BiOCl, and polypyrrole, and explores their integration with unconventional water sources such as industrial brines, hypersaline reject streams, and treated wastewater, particularly within the resource-constrained context of the Atacama Desert. This manuscript consolidates experimental data, device designs, and comparative performance metrics, providing a critical framework for advancing blue energy technologies. It also underscores their potential role in circular economy models and off-grid renewable energy systems solutions.

## 1 Introduction

Chile, particularly its northern macrozone, offers a highly favorable scenario for the deployment of salinity gradient energy technologies, due to its unique geographic, industrial, and hydrological characteristics. The Atacama Desert is the site of significant brine extraction activities, which generate large volumes of concentrated liquid waste, particularly rich in lithium. By combining these residual brines with other water sources, such as seawater, desalination plant wastewater, or municipal wastewater, it is possible to obtain media with salinity gradients suitable for energy recovery applications (Cabello, 2021; Fitzsimons and Warren, 2024; He et al., 2024; Kidder et al., 2020; Lagos et al., 2024; Marinova et al., 2025). These contrasting water streams establish naturally available, high-enthalpy salinity gradients that are ideal for electrochemical energy harvesting, particularly via Mixing Entropy Battery (MEB) technology. Compared to other technologies such as (i) electrodialysis (RED) (Ahualli et al., 2014; Brogioli, 2009; Jia et al., 2013; Jia et al., 2013; Kim et al., 2016a; Kim et al., 2016b; La Mantia et al., 2011; Lee et al., 2017; Logan and Elimelech, 2012; Logan and Elimelech, 2012; Marino et al., 2014; Md Hasan et al., 2017), (ii) Pressure Retarded Osmosis (PRO) (Ahualli et al., 2014; Bag, 2017; Haj Mohammad Hosein Tehrani et al., 2015; Jia et al., 2014a; Ye et al., 2014), (iii) semipermeable membranes (Brogioli, 2009; Brogioli et al., 2012; La Mantia et al., 2011), (iv) selective ion membranes (Bag, 2017; Brogioli, 2009; La Mantia et al., 2011) and (v) concentration cells (Bag, 2017; Brogioli, 2009; Jia et al., 2013; La Mantia et al., 2011), MEBs offer a simpler system capable of operating efficiently in high-saline environments. The exponential increase in global energy demand, along with the detrimental environmental impact caused by fossil fuel consumption, has necessitated the exploration of sustainable alternatives derived from abundant natural resources. This change has redirected attention away from petroleum-based fuels toward cleaner and renewable energy sources. Among the most successful alternatives to date are solar, wind and geothermal energy (Chang et al., 2023; Gómez et al., 2025; Osorio-Aravena et al., 2021; Osorio-Aravena et al., 2025; Oyarzún-Aravena et al., 2025; Véliz et al., 2025). However, the vast expanse of the ocean remains an underexploited and inexhaustible reservoir of renewable energy, which is a relevant opportunity for Chile, given its extensive Pacific coastline that spans the entire length of the country. Marine energy sources, including waves, tides, ocean currents, offshore winds, thermal gradients, and salinity concentration differences, have emerged promising avenues for sustainable energy generation (Marino et al., 2015). In particular, the chemical energy released during the natural mixing of freshwater and seawater at estuarine interfaces, commonly referred as blue energy or salinity gradient energy (Lee et al., 2017; Marino et al., 2014), presents a unique opportunity for electricity generation. This entropic energy release, which occurs when river water meets seawater flow, is estimated to yield up to 2.2 kJ of free energy per liter of freshwater (Brogioli, 2009; Brogioli et al., 2012; Kim et al., 2016a; Kim et al., 2016b; La Mantia et al., 2011; Marino et al., 2015; Md Hasan et al., 2017; Morais et al., 2016). Despite the potential application of MEBs, the use of the hypersaline electrolytes from Atacama Desert and their associated corrosion challenges remain poorly studied.

In 1954, Pattle was the first to propose the generation of renewable energy through the mixing using of freshwater with water of higher ionic concentration, introducing it as an alternative to generate clean energy (Pattle, 1954; Fernández et al., 2015). Later, in 1976, Bert H. Clampitt et al. proposed an electrochemical cell that could recover energy using the concept of mixing waters of different concentrations, as river water and seawater (Clampitt and Kiviat, et al., 1976). In 2009, Brogioli (2009) explained a new and interesting technique of obtaining blue energy under the electrochemistry concept called CAPMIX (Brogioli et al., 2012; Fernández et al., 2015; Gomes et al., 2015; Haj Mohammad Hosein Tehrani et al., 2015; Iglesias et al., 2014; Jia et al., 2013; Jia et al., 2014a; Lee et al., 2017; Marino et al., 2014; Marino et al., 2016; Ye et al., 2014), based on the storage of Na^+^ and Cl^−^ ion inside activated carbon electrodes (Brogioli, 2009; Brogioli et al., 2012; Fernández et al., 2015; Iglesias et al., 2014; Jia et al., 2014a; Lima et al., 2017; Marino et al., 2015; Marino et al., 2016). This technology has technical disadvantages due to problems to sensitivity to impurities such as dissolved oxygen during the mixing solutions (La Mantia et al., 2011; Salerno et al., 2013). These impurities generate uncontrolled discharges (Brogioli, 2009; La Mantia et al., 2011; Marino et al., 2016) which makes entropic energy production inefficient. In 2011, Fabio La Mantia et al. (La Mantia et al., 2011) proposed a revolutionary system, obtaining called Mixing Entropy Battery (MEB). This technology generated expectations and advanced the technology by working similarly to Brogioli device, utilizing the mixture of two solutions with varying salinity concentrations. The MEB functions as a reversible electrochemical system, causing electroactive ions in the solution with high ionic strength to be stored pseudo-capacitively into the crystalline structure of the cathode and anode materials, respectively (Ali et al., 2025; Du et al., 2025; Li et al., 2025a; Li et al., 2025b; Wang et al., 2025b). The CAPMIX and MEB signify significant strides in energy storage technology. This manuscript compiles relevant information on various electroactive materials that employ the technology and concept of electrochemical ion pumping method for blue energy recovery, with a particular focus on their potential application in the Atacama Desert, Chile.

## 2 Opportunity of Chile for blue energy recovery

La Mantia reported a significant advancement in blue energy using LiCl as the electrolyte, which is an approach particularly relevant to lithium-rich brines from the Atacama Desert. The alternative electrochemical system operates based on the reversible reaction FePO_4_ + Ag^+^ + LiCl ⇌ LiFePO_4_ + AgCl. In this configuration, the LiFePO_4_/FePO_4_ was employed as cathodic electrode (facilitating lithium-ion intercalation), while the Ag/AgCl was employed as anodic electrode (enabling chloride ion capture). Lithium chloride (LiCl), which is highly soluble in water (832 g/L), enables the generation of steep salinity gradients favorable for efficient energy extraction. To simulate freshwater and seawater conditions, LiCl concentrations of 0.03 and 1.5 M were employed, respectively. The system achieved an energy density of 38.2 mJ/cm^2^ and a power density of 13.8 μW/cm^2^, slightly surpassing the performance of the NaCl/Na_2_Mn_5_O_10_ system. Furthermore, it demonstrated excellent cycling stability over 100 cycles, with no observable decline in energy output. The cell voltage remained stable throughout operation, attributed to the two-phase nature of the LiFePO_4_/FePO_4_ electrode pair (La Mantia et al., 2011). Northern Chile offers a unique combination of environmental and industrial conditions that position it as a prime candidate for the deployment of salinity gradient energy recovery technologies using the saline waters present in the Atacama Desert. The region is characterized by substantial volumes of high-salinity effluents generated by industrial activities, such as lithium brine obtained via evaporation of naturally well-brine (Foo and Lienhard, 2025; Gutiérrez and Ruiz-León, 2024; He et al., 2024; Marinova et al., 2025). These concentrated streams abruptly contrast sharply with low-salinity sources such as municipal wastewater (Furness et al., 2024; Phuc-Hanh Tran et al., 2024; Sampedro et al., 2024; Soo et al., 2024; Zhan et al., 2025), creating favorable salinity gradients that are ideal for blue energy recovery through electrochemical technologies. A promising opportunity lies along the coastal interface of the Atacama Desert, where treated effluents from desalination plants or municipal wastewater facilities (de Lima et al., 2025; Giacalone et al., 2024) can be mixed with hypersaline residual brines from industries as SQM, Albemarle, or future operations by Codelco, forming a gradient with entropic potential exceeding that of natural seawater/river water systems (Cabello, 2021; Lagos et al., 2024; Marinova et al., 2025). From a technological perspective, Chile’s lithium-rich brines are compatible with Li-selective cathodes such as LiFePO_4_, enabling dual functionalities: (i) energy extraction, and (ii) lithium pre-concentration and recovery (Liu et al., 2025; Lv et al., 2025; Ou et al., 2024; Zhang et al., 2024; Zhang et al., 2025). Similarly, CuHCF materials could be synthetized using low-cost, locally available precursors derived from salts such as NaNO_3_, KNO_3_, NaCl, KCl, CuSO_4_, among other located in the Atacama Desert from Lithium and fertilizer industries. Furthermore, the Atacama Desert’s exceptionally high solar irradiance (exceeding 3,000 kWh/m^2^ year) (Bayo-Besteiro et al., 2023; Luccini et al., 2016; Marzo et al., 2018; Soler et al., 2025) enables the integration of solar thermal systems for water recovery via solar distillation, solar concentration or photovoltaic-powered brine reuse, creating a hybrid solar-blue energy loop with net-zero emissions.

## 3 Materials for blue energy recovery

Research into blue energy has traditionally focused on the mixing of seawater and freshwater. However, alternative sources, such as highland brines, municipal wastewater, or reject saline solutions from reverse osmosis plants, are gaining high interest as viable salinity gradient media. The use of commercial cathode materials commonly employed in lithium-ion batteries allows for the reversible intercalation of Na^+^ or Li^+^ ions, enabling Faradaic reactions driven by salinity gradients (Altiok et al., 2023; Gaber et al., 2025; Galleguillos et al., 2020; Galleguillos-Madrid et al., 2024; Liu et al., 2022; Mojid et al., 2024; Salazar-Avalos et al., 2023; Suu et al., 2025).

Despite their high energy efficiency, the use of silver (Ag) as an anode is limited due to its high cost and strong thermodynamic susceptibility to form AgCl layers in chloride-containing media (Chauhan et al., 2025; De Silva et al., 2011; Vvedenskii et al., 2007), as well as its tendency to form complexes with ions such as NH_4_^+^ and CO_3_^2-^ (Cho et al., 2022; Jin et al., 2003; Pargar et al., 2018; Popović et al., 2023). In a simulated NaCl gradient (0.6 vs. 0.024 M NaCl), energy recovery values of up to 29 mJ/cm^2^ have been reported with 75% efficiency, while under a LiCl gradient (1.5 vs. 0.03 M LiCl), recoveries of 38.2 mJ/cm^2^ have been achieved over 100 cycles (La Mantia et al., 2011). Jia et al. (2015) presented a system using CuHCF as the anode and Ag as the cathode, demonstrating reversible Na^+^ intercalation/deintercalation within the CuHCF crystal structure. Kasiri et al. (2019) proposed the use of CuZnHCF mixture as a cathode material in aqueous zinc-ion batteries, achieving capacity retention of 85.54% at 1C after 1,000 cycles. Similarly, Haj Mohammad Hosein Tehrani et al. (2015) utilized CoHCF as the cathode, observing two redox peaks associated with the Fe^2+^/Fe^3+^ coupled and Na^+^ ion transport, and achieving an energy recovery of up to 24,000 μW/g. In a related study, Lu et al. (2016) evaluated the use of CoHCF as a cathode in sodium-based energy conversion systems, showing excellent capacitance and high specific energy of 54.4 Wh/kg. Kim et al., (2016a) proposed the use of NH_4_HCO_3_ as a low-temperature dissociable salt, allowing the operation of hybrid electrochemical cells that integrate thermal distillation. In this configuration, MnO_2_ showed stability in the presence of NH_4_^+^, while PbO and PbO_2_ were evaluated as anodic electrode in CO_3_^2-^ containing electrolytes, however, the formation of PbCO_3_ on PbO surfaces limits efficiency during the operation. The Hybrid CapMix concept, introduced by Lee et al. (2017), combines a Faradaic cathode (sodium manganese oxide, NMO) with an activated carbon as anode, and an anion exchange membrane, achieving a power density of 97 mW/m^2^. Likewise, Kim et al. (2016b) demonstrated the viability of using non-selective membranes as low-cost alternatives to ion-exchange membranes, producing stable power outputs of 411 mW/m^2^ over 20 cycles. Ye et al. (2019) proposed a MEB using Prussian Blue (PB) and Polypyrrole (PPy), both low-cost materials. The device exhibited nearly 100% Coulombic efficiency and stable operation over 50 cycles, demonstrating the feasibility of energy-free operation. Tan and Zhu (2020) introduced BiOCl as a cost-effective alternative to Ag, achieving power densities up to 87 mW/m^2^ with the aid of polyelectrolyte coatings. The system exhibited good stability in hypersaline waters (300 g/L NaCl), offering results comparable to or exceeding those of AgCl-based systems. Nevertheless, the use of Ag remains problematic due to partial dissolution in real-world environments, with detected concentrations exceeding EPA limits after several cycles (Ye et al., 2014). Therefore, the development of sustainable materials such as PPy or BiOCl, are essential for the scalable deploying of this technology.

## 4 Discussion and outlooks

Currently, several techniques are available to recover chemical energy from the salinity gradient between two solutions, including reverse electrodialysis (RED) (Ahualli et al., 2014; Brogioli, 2009; Jia et al., 2013; Kim et al., 2016a; Kim et al., 2016b; La Mantia et al., 2011; Lee et al., 2017; Logan and Elimelech, 2012; Marino et al., 2014; Md Hasan et al., 2017), Pressure Retarded Osmosis (PRO) (Ahualli et al., 2014; Bag, 2017; Haj Mohammad Hosein Tehrani et al., 2015; Jia et al., 2014b; Ye et al., 2014), semipermeable membranes (Brogioli, 2009; Brogioli et al., 2012; La Mantia et al., 2011), selective ion membranes (Bag, 2017; Brogioli, 2009; La Mantia et al., 2011), and concentration cells (Bag, 2017; Brogioli, 2009; Jia et al., 2013; La Mantia et al., 2011). All these technologies have undergone significant technological advancements in recent years. RED systems, which operate using ion-exchange membranes, typically achieve power densities in the range of 1–2 W/m^2^ and perform more efficiently under low salinity gradients. This technology can be integrated to other processes, such as reverse osmosis; however, the cost associated with building, operation and membrane replacement can be limitations to widespread adoption. In contrast, PRO is better suited for high salinity gradients but requires high-pressure-resistant membranes and complex infrastructure. This technology commonly achieves power densities around of 5–7 W/m^2^ using seawater/wastewater, and 27 W/m^2^ using 1 M NaCl with distilled water (Wan and Chung, 2015). Mixing Entropy Batteries (MEBs), however, operate without membranes or rely on simple separators, enabling energy recovery from highly concentrated brines while significantly reducing system complexity. Although current MEBs yield lower power densities (typically between 10 and 100 mW/m^2^), they offer substantial advantages and versatility in terms of electrode material, modular design, and compatibility with industrial effluents, features particularly beneficial in resource-limited or remote regions such as the Atacama Desert. Furthermore, both RED and PRO are heavily reliant on membranes, for which sustainable recycling or circular economy strategies are not yet widely implemented. In contrast, MEBs adopt a membrane-free concept that facilitates direct electrochemical energy recovery during the mixing of waters with differing salinity.

The strategic convergence of industrial activity, geographical conditions, and saline water resource dynamics in Chile, especially in the Atacama Desert, creates a favorable environment for the development of salinity gradient energy recovery technologies. Brine mining operations not only generate massive quantities of high-salinity effluents, especially lithium-rich brines, but are also complemented by the availability of desalinated seawater and municipal wastewater to meet operational demands in regions of high solar irradiance (Ali Chang et al., 2024; de Lima et al., 2025). These diverse water sources naturally create sharp ionic gradients, which can be connected for blue energy generation using electrochemical technologies such as Mixing Entropy Batteries (MEBs) or hybrid CapMix systems (Cheng et al., 2025; El Moutchou et al., 2024). Chile presents a scenario of high-enthalpy salinity gradients. Industrial brines in northern Chile often exceed 200–300 g/L NaCl (Bonelli and Pavez, 2025; He et al., 2024). Additionally, Chile’s mining sector directly contributes the advancement of blue energy systems. For example, lithium-rich brines match well with Li-selective cathodes like LiFePO_4_ or NiHCF (Goel et al., 2025; Wei et al., 2025; Xu et al., 2023), enabling simultaneous energy harvesting and lithium pre-concentration. Similarly, materials such as CuHCF and PPy, which are promising candidates for cathode and anode materials, could be synthesized from local resources such as NaNO_3_ or KNO_3_ from SQM’s operations. This presents a novel opportunity to deploy emerging technologies that can contribute power generation in hard-to-reach regions such as the Chilean Altiplano. From a technological perspective, a wide range of materials have been evaluated for their suitability in electrochemical systems exploiting salinity gradients. Cathodes, such as NMO and lithium-iron phosphate (LFP), enable Faradaic ion intercalation reactions with Na^+^ and Li^+^, respectively, offering high specific capacities and stability. However, the widespread use of silver (Ag) as an anode raises sustainability concerns due to its cost and solubility issues in high-chloride environments, resulting in the formation of AgCl or complex ions that impair system efficiency (Man et al., 2025; Suu et al., 2025; Yim et al., 2025).

The experimental performance of MEBs has been validated through various configurations reported in the literature, as summarized in Table 1. These systems demonstrate successful operation under laboratory conditions simulating real-world salinity gradients. For example, the Na_2_Mn_5_O_10_–Ag and LiFePO_4_–Ag configurations (La Mantia et al., 2011) achieved energy recovery values of 10.5 and 13.8 μW/cm^2^, respectively, under synthetic gradients (0.6 vs. 0.024–0.03 M NaCl or LiCl) with stable cycling over 100 cycles. Other systems, such as Na_4_Mn_9_O_18_–Ag (Jia et al., 2014a; Jia et al., 2014b) and CoHCF–Ag (Kiviat, 1976) have reported power densities of up to 0.65 kW/m^3^ and 24 mW/g^1^, respectively, confirming their electrochemical reversibility and robustness. Additionally, Faradaic systems based on CuHCF or BiOCl (Logan and Elimelech, 2012; Ye et al., 2019) have demonstrated acceptable energy recovery ranging from 10 to 411 mW/m^2^, even in configurations with minimal electrode areas or simplified geometries. Collectively, these findings provide a solid experimental foundation for the viability of MEBs and support the conceptual projections outlined in this work. While laboratory results are promising, long-term stability of electrode materials under real brine conditions remains a key challenge for scale-up. Several anodes reported in the literature, such as silver (Ag), are prone to degradation due to chloride-induced reaction (AgCl layer formation), particularly in high-chloride industrial brines. This issue is evident in multiple studies (Fernández et al., 2015; La Mantia et al., 2011; Ye et al., 2014), where Ag is used despite its known environmental risks and tendency to dissolution under prolonged cycling. Alternative anodes such as BiOCl and PPy have emerged as promising substitutes due to their chemical stability and lower environmental impact (Marino et al., 2016; Ye et al., 2019). On the cathodic side, materials like LiFePO_4_, Na_2_Mn_5_O_10_, and CuHCF have demonstrated high reversibility; however, their performance can be influenced by pH, ionic composition, and the presence of complexing agents such as NH_4_^+^ or CO_3_^2-^. Additionally, the pond of standardized water pre-treatment protocols introduces uncertainty in systems that rely on municipal wastewater or desalination brines. Membrane-based systems such as hybrid CapMix, PRO or RED are further limited by membrane fouling and degradation under hypersaline and organic-rich conditions issues that remain unresolved in the absence of recycling strategies or circular economy models.

**TABLE 1:** Characteristics of the mixing entropic batteries–design and operations conditions described in the literature.

Ref	Cathodic material	Anodic material	Electrode area (cm^2^)	Thickness electrode (mm)	MEB volume (cm^3^)	Electrode spacing (mm)	Potential range (V)	Current range (mA/cm^2^)	High concentration solution (M)	Low concentration solution (M)	Cycles No	Recovery potential (V)	Efficiency (%)	Recovered energy W/m^2^
La Mantia et al. (2011)	Na_2_Mn_5_O_10_	Ag	2	—	0.35	10	—	± 0.25	0.6	0.024	100	0.135	75	0.105
La Mantia et al. (2011)	LiFePO_4_	Ag	2	—	0.35	10	—	—	1.5	0.030	100	—	—	0.138
Md Hasan et al. (2017)	Na_2_Mn_5_O_10_	Ag	2	—	0.30	1	0–0.65	± 0.50	0.6	—	—	—	—	—
Ye et al. (2014)	Zn	Ag	1	0.1	—	0.6	0.8–1	± 0.50	4.5%	0.68	—	0.160	80	1.6
Marino et al. (2016)	NiHCF	Ppy/Ag	1	—	—	—	0–0.8	± 0.01	3	0.020	100	—	—	—
Salerno et al. (2013)	Na_2_Mn_5_O_10_	AC	3.01	0.3	0.06	0.2	0–0.6	± 0.5	0.6	0.01	3	0.150	—	0.097
Trocoli et al. (2016)	MnO_2_	Pb	7	0.2	14.1	—	0.1–0.8	± 0.03	1	0.020	—	0.100	—	0.0063
Logan and Elimelech (2012)	Ag	CuHCF	5	—	—	3	0.2–1.2	± 0.50	0.6	0.024	25	0.102	69	17.95
Jia et al. (2014a)	Na_4_Mn_9_O_18_	Ag	9	1.7	1.5	1.7	—	± 0.25	0.6	0.032	12	—	68	650
Fernández et al. (2015)	Na_2_Mn_5_O_10_	Ag	1	0.05	—	0.6	—	—	0.5	0.020	—	—	—	0.015
Kiviat (1976)	CoHCF	Ag	1.3	—	—	1	0–1.1	± 0.01	0.6	0.024	30	0.153	65	—
Pasta et al. (2012)	CuHCF	CuHCF	3	0.12	1.2	0.4	—	± 0.5	0.513	0.017	20	0.172	—	0.411
Ye et al. (2019)	FeHCF	PPy	—	—	—	—	0–0.3	—	0.6	0.024	50	0.3	—	—
Tan and Zhu (2020)	CuHCF	BiOCl	—	—	—	—	0.5	0.2	5.14	0.017	—	—	—	0.010
Smolinska-Kempisty et al. (2020)	C	C	0.2	0.2	—	—	0.2	1,380	1.71	0.069	10	—	86.3	0.017
Nasir et al. (2020)	C	C	9	4.5	—	1	0.6	—	0.5	0.001	1	0.6	—	—
Li et al. (2023)	MoS_2_	C	1	—	—	—	0.4	5	0.086	—	100	—	82.2	0.00612

[a] NaCl concentrated solution resistance: 5 Ω, resistance of NaCl diluted solution: 75 Ω [b] LiCl concentrated solution resistance: 5.4 Ω, resistance of the diluted LiCl solution: 10.6 Ω, [c] Load capacity of Na_2_Mn_5_Or_10_ is 35 mAh/g, [d] NaCl concentrated solution resistivity: 0.1 Ωm, NaCl diluted solution resistivity: 1.3 Ωm and a solution feed flow of 5 mL/s, [e] NaCl concentrated solution resistance: 3 Ω, resistance of NaCl diluted solution: 17 Ω and load capacity for Na_2_Mn_5_O_10_ material of 35 mAh/g, [f] 0.5 mL/s, [g] The energy obtained is based to each kJ/mol of Na^+^ electro inserted into each charge and discharge cycle, [h] The MEB prototype used considers a cation exchange membrane, [i] Resistance of NaCl concentrated solution: 0.3 Ω, resistance of NaCl diluted solution: 5.6 Ω, membrane resistance: 1.0 Ω, solution feed flow: 7.5 mL/min, [j] Activated carbon (AC), anionic exchange membrane with an area of 3.01 cm^2^, solution feed flow: 20 mL/min.

MEBs offer a membrane-free or minimal-membrane alternative, thereby reducing maintenance burdens and reducing the need for intensive chemical conditioning. However, ensuring stable performance in real effluents streams will require the development of tailored pre-treatment strategies, such as particulate filtration, anti-scalants, or ultraviolet (UV) disinfection, depending on the specific characteristics of the water source. Consequently, further pilot-scale studies are essential to evaluate fouling resistance, redox stability, and long-term durability of MEB electrodes operating in complex saline matrices.

Research has shown that Ag concentrations in treated water can exceed regulatory limits, such as those set by the US EPA, after multiple-discharge cycles (Cervantes-Avilés et al., 2019; Shafer et al., 1998; Wimmer et al., 2019). As a sustainable alternative, BiOCl has demonstrated energy densities comparable to Ag-based systems, reaching up to 87 mW/m^2^ when enhanced with polyelectrolyte coatings (Dhanasekaran et al., 2025; Reale et al., 2021; Zhou et al., 2022). Similarly, PPy as a conducting polymer, exhibits robust performance, broad potential windows, and low energy input requirements, making it well-suited for the selective capture of anions such as Cl^−^. Transition metal oxides like MnO_2_ also show high redox stability, particularly in systems utilizing ammonium bicarbonate (NH_4_HCO_3_) as a regenerative salt for thermal-electrochemical hybrid configurations (Chen et al., 2025; Jabarullakhan and Kandasamy, 2025; Meng et al., 2020; Wang et al., 2025a). Moreover, research on hybrid CapMix systems has demonstrated promising performance under real-world conditions. These devices, that combine capacitive and Faradaic charge storage strategies, have achieved energy recoveries values approaching 100 mW/m^2^ under moderate salinity gradients. Notably, Lee et al. (2017) reported a hybrid device based on NMO and activated carbon that exemplifies this synergistic behavior. Additionally, the use of non-selective membranes in place of costly ion-exchange membranes, as demonstrated by Kim et al. (2016a) and Kim et al. (2016b), provides a practical path for large-scale implementation.

Importantly, Chile’s exceptionally high solar irradiance, surpassing 3,000 kWh/m^2^·year in regions like the Atacama Desert (Luccini et al., 2016; Marzo et al., 2018; Naranjo et al., 2025; Rodríguez-Córdova et al., 2025), offers a strategic advantage for the integration of solar-driven or hybrid energy systems. Looking forward, the implementation of blue energy in Chile can help address several national priorities: (i) diversifying the energy matrix, (ii) valorizing industrial waste streams, and (iii) decarbonizing energy-intensive sectors. By leveraging its lithium and copper resources not only for global battery markets but also for electrochemical blue energy systems, Chile has the potential to pioneer integrated circular economy models that promote clean energy innovation. However, the long-term performance and fouling resistance of electrode materials under real industrial brine conditions must be rigorously evaluated (Arif et al., 2017; Liu et al., 2014; Yangyang Wang, 2022; Yue et al., 2025). Issues such as membrane degradation, ionic selectivity, and cycling efficiency under complex effluent compositions also remain unresolved and demand further study. Furthermore, techno-economic analyses that incorporate site-specific variables, including local flow rates of waters, salinity profiles, and capital and operational expenditures, are essentials to determinate the scalability and investment feasibility of proposed systems.

The exploration of blue energy as a viable alternative to conventional renewables marks a pivotal step toward the global pursuit of sustainable and resilient energy systems. Among the most promising approaches, electrochemical methods for harvesting entropic energy at the interface of fluids with differing salinities, whether natural or anthropogenic origin, have demonstrated robustness and adaptability. In this context, Chile is uniquely positioned to pioneer the industrial-scale implementation and development of blue energy systems. Its northern macrozone, with high lithium and copper resources, abundant access to high-salinity industrial brines, and desalinated or treated water, offers an exceptional salinity gradient that can exceed even that found at natural seawater-river water interfaces. These conditions not only enable efficient blue energy generation with high efficiency but also create opportunities to value critical mineral residues and close the loop on water-energy nexus challenges. On the other hand, the use and deployment of low-cost and sustainable electrodes such as BiOCl, MnO_2_, CuHCF, or Polypyrrole (PPy), alongside innovative configurations that integrate Faradaic and capacitive charge storage, has enabled stable and scalable energy recovery across a broad range of saline mixtures. Importantly, many of these materials are increasingly compatible with local Chilean resources, particularly lithium-rich brines, offering new avenues for national innovation and industrial cooperation. Moreover, coupling blue energy technologies with Chile’s exceptional solar potential opens the door to hybrid systems in which solar heat is used for distillation or concentration of saline solutions, enhancing system autonomy and net energy yield with zero emissions. Ultimately, blue energy stands as a promising technology for diversifying renewable energy sources. Its capacity to extract chemical energy from water salinity gradients, even under extreme salinity conditions, provides a solid scientific and technological foundation for large-scale deployment. Moving forward, coordinated efforts between academia, industry, and government will be essential to transition from laboratory-scale demonstrations to commercially viable, field-ready solutions. Such collaborations will ensure that blue energy not only contributes to mitigating climate change but also fosters innovation, water circularity, and sustainable development of clean energy.

The implementation of blue energy in Chile holds transformative impact across industrial, economic, social, and environmental dimensions. At the industrial scale, this technology represents a critical step toward the decarbonization of key processes, enabling a more diversified and resilient energy matrix while significantly reducing dependence on fossil fuels. These advances will enhance national energy security and contribute to a substantial reduction in our carbon footprint. For small and medium-sized enterprises (SME), the development of blue energy opens new market opportunities in the design, manufacture, installation, and maintenance of MEB systems. These ecosystems will foster the growth of specialized technical skills, facilitate knowledge transfer, and generate a wide range of skilled employment opportunities, simulation local economies and promoting technological sovereignty. Socially, blue energy offers a unique opportunity to improve the lives of remote communities in regions such as the Atacama Desert, where access to reliable electricity remains a challenge. By utilizing locally available water resources, blue energy systems can support models of regional energy autonomy, enhancing living conditions, and advance sustainable rural development. Strategically, this initiative positions Chile as a frontrunner in global energy innovation. In doing so, Chile reinforces its leadership in the global energy transition, advancing both environmental and economic competitiveness.

## Data Availability

The datasets presented in this article are not readily available because there is no new data. The article considers previously published articles. Requests to access the datasets should be directed to Felipe Galleguillos/felipe.galleguillos.madrid@uantof.cl.
